# Nanopore Sequencing for Detection and Characterization of Phosphorothioate Modifications in Native DNA Sequences

**DOI:** 10.3389/fmicb.2022.871937

**Published:** 2022-04-21

**Authors:** Taylor Wadley, Sun Hee Moon, Michael S. DeMott, Visanu Wanchai, En Huang, Peter C. Dedon, Gunnar Boysen, Intawat Nookaew

**Affiliations:** ^1^Department of Biomedical Informatics, University of Arkansas for Medical Sciences, Little Rock, AR, United States; ^2^Department of Pathobiology, University of Arkansas for Medical Sciences, Little Rock, AR, United States; ^3^Department of Environmental and Occupational Health, University of Arkansas for Medical Sciences, Little Rock, AR, United States; ^4^Department of Biological Engineering, Massachusetts Institute of Technology, Cambridge, MA, United States; ^5^Winthrop P. Rockefeller Cancer Institute, University of Arkansas for Medical Sciences, Little Rock, AR, United States

**Keywords:** native sequence, phosphorothioate modifications, *Salmonella enterica*, *dnd* operon, nanopore, ELIGOS

## Abstract

Bacterial DNA is subject to various modifications involved in gene regulation and defense against bacteriophage attacks. Phosphorothioate (PT) modifications are protective modifications in which the non-bridging oxygen in the DNA phosphate backbone is replaced with a sulfur atom. Here, we expand third-generation sequencing techniques to allow for the sequence-specific mapping of DNA modifications by demonstrating the application of Oxford Nanopore Technologies (ONT) and the ELIGOS software package for site-specific detection and characterization of PT modifications. The ONT/ELIGOS platform accurately detected PT modifications in a plasmid carrying synthetic PT modifications. Subsequently, studies were extended to the genome-wide mapping of PT modifications in the *Salmonella enterica* genomes within the wild-type strain and strains lacking the PT regulatory gene *dndB* (*ΔdndB*) or the PT synthetic gene *dndC* (*ΔdndC*). PT site-specific signatures were observed in the established motifs of GAAC/GTTC. The PT site locations were in close agreement with PT sites previously identified using the Nick-seq technique. Compared to the wild-type strain, the number of PT modifications are 1.8-fold higher in *ΔdndB* and 25-fold lower in *ΔdndC*, again consistent with known regulation of the *dnd* operon. These results demonstrate the suitability of the ONT platform for accurate detection and identification of the unusual PT backbone modifications in native genome sequences.

## Introduction

DNA is modified with different chemical groups that bestow it with different properties, such as protection from enzymatic digestion and regulation of gene expression. One form of DNA modification found in nearly all types of bacteria and archaea is the phosphorothioate (PT). A PT modification involves a sulfur atom replacing a non-bridging oxygen in the DNA phosphate backbone. It is a naturally occurring DNA modification in many bacterial and archaeal species (Wang et al., 2007). The PT modification is known to protect DNA against restriction endonuclease activity (Tong et al. *in press*). The operon was originally termed the DNA degradation operon (*dnd*) due to chemically induced strand breaks at PTs during electrophoresis. A 4-5 gene *dnd* cluster encodes the enzymes that produce and regulate PT modifications, often accompanied by a 3-4 gene cluster encoding a restriction system (Tong et al., 2018).

The *dnd* operon functions as part of the prokaryotic innate immune system to protect the cells from foreign DNA (Xu et al., 2010). The *dndBCDE* genes encode the enzymes that modify the DNA backbone. The sequence motif preferable for the enzymes varies by species, with *Escherichia coli* B7A and *Salmonella enterica* subspecies *enterica* serovar Cerro 87 (GCA_001941405.1_ASM194140v1) sharing the 5′-G_PS_AAC-3′/5′-G_PS_TTC-3′ motif. The *dndFGH* genes (Wang et al., 2007) encode for the restriction enzyme system. The presence of PT modifications is also beneficial for the microbe to protect the DNA against some types of oxidative stress (Tong et al., 2018).

*Salmonella enterica* serovar Cerro 87 contains the complete operon with *dndABCDE* and *dndFGHI* genes (He et al., 2015). DndB is a regulatory protein that controls PT levels by binding to promoter regions of the operon, and deletion of *dndB* caused an upregulation of PT modifications (Wang et al., 2019). The *dndACDE* genes encode the proteins (DndACDE) that form a large enzyme complex that replaces a non-bridging oxygen with the sulfur modification (Xiong et al., 2015). DndA provides the sulfur atom, which is transferred to DndC, while DndD nicks the phosphodiester bond, with DndE stabilizing the nicked strands. Interfering with this enzyme complex downregulates PT modifications (Wang et al., 2019).

The first glimpse into the genetic context of PT modifications was accomplished using liquid chromatography (LC)-mass spectrometry (MS). It showed three discrete genomic frequencies resulting in PT specific phenotypes in various bacterial species (Wang et al., 2011). LC–MS/MS is a good tool for establishing which DNA modifications are present; however, it cannot show where in the genome these PT modifications are located. Sequencing DNA to uncover modifications was not possible until the emergence of third-generation sequencing platforms (Simpson et al., 2017). Genomic mapping of PT modifications was first performed by Cao et al. (2020) using two different sequencing techniques (Cao et al., 2014). The first technique used SMRT sequencing (Pacific Biosciences of California, Inc.) that takes advantage of the interpulse duration of the DNA polymerase kinetics to detect DNA modifications natively. The second technique, Nick-seq, uses iodine to induce DNA strand breaks at PT sites followed by Illumina Next-Generation Sequencing of resulting DNA fragments to localize PT sites through bioinformatic analysis (Cao et al., 2020).

We recently demonstrated that Oxford Nanopore Technologies (ONT), which is a third-generation sequencing platform can detect nucleic acid modifications (Jenjaroenpun et al., 2020; Nookaew et al., 2020; Boysen and Nookaew, 2022). The ONT platform sequences native DNA and RNA, with nucleotide bases having their own electrical signal known as a “squiggle.” The ONT platform can detect modified bases because of the difference in the squiggle between modified and unmodified bases (Xu and Seki, 2020). To our best knowledge, it has not been applied for detection of DNA backbone modifications, such as PT.

This study aimed to demonstrate the suitability of the ONT platform to identify and characterize PT modifications in DNA. We report herein the detection of PT modification by epitranscriptional landscape inferring from glitches of ONT signals (ELIGOS) using synthetic PT modifications and native DNA from *S. enterica* containing naturally occurring PT modifications in wild-type, *dndB* knockout (∆*dndB*) mutant (known to increase PT modifications), and *dndC* knockout (∆*dndC*) mutant (known to decrease PT modifications). The ONT platform of DNA sequencing identified the PT modifications from native long-read sequences, and the results agree with previously reported PT sites in the same three *S. enterica* strains.

## Materials and Methods

### PT Oligonucleotide Construction and Insertion Into pUC19 for Sequencing

To verify that the ONT platform can detect PT modifications, we designed a pUC19 plasmid vector harboring a synthetic 98-basepair 2′deoxyoligonucleotide carrying known PT modification motifs (Supplementary Figure S1).

To create the plasmid with the PT modifications, the following was performed as: (1) the synthetic 2′deoxyoligonucleotide was designed with sticky ends (a four-nucleotide overhang on the positive strand) that complemented the cut sites of the XbaI and KpnI endonucleases and four PT modification sites; (2) the pUC19 plasmid was transformed into *E. coli* methyltransferase-deficient *dam*^−^/*dcm*^−^ competent cells (NEB) following the High-Efficiency Transformation Protocol (NEB) and grown overnight at 37°C in tryptic soy broth supplemented with ampicillin; then, the plasmid DNA was extracted using the Monarch Plasmid Miniprep Kit (NEB) and treated with the endonucleases to cut the plasmids, XbaI at position 656 and KpnI at position 675, to generate sticky ends; (3) to maximize ligation yield, the cut plasmid was incubated with a concentration (4×) of the synthetic 2′deoxyoligonucleotide and T4 DNA ligase (NEB) overnight at 16°C; and (4) the ligation was validated by running the ligated plasmid, pUC19_pt, with the control plasmid, pUC19, on 2% agarose gel for 2 h at 100 volts (Supplementary Figure S2).

### Plasmid Transformation of pUC19_PT to Generate a PT Modification-Free Plasmid

Approximately 100 ng of the pUC19_pt plasmid was extracted from an agarose gel and cleaned using the Monarch DNA Gel Extraction Kit (NEB) according to the manufacturer protocol. The plasmid was then transformed into the methyltransferase-deficient *dam*^−^/*dcm*^−^ competent *E. coli* cells (NEB) as described in detail above to create a modification-free control plasmid lacking the PT modifications (PT modification-free plasmid).

### Plasmid Library Preparation and Sequencing

The pUC19_pt plasmids and PT modification-free plasmids were used as the input for the sequencing library preparation using the Rapid Barcoding Kit (ONT). Two samples (~400 ng each) for each of the two plasmids were used for the sequencing library construction, according to the manufacturer protocol. Sequencing was performed on a single R9.4 FLO-MIN106 flow cell on a MinION Mk1B for 24 h through MinKNOW software to generate fast5 files.

### Bacterial Strains and Cultivation

The wild-type (dnd+) *S. enterica* serovar Cerro 87 strain was originally provided by Dr. Toshiyuki Murase at Tottori University, Japan (Murase et al., 2004). The mutant strains, *S. enterica* YF10 (*ΔdndB*) and YF11 (*ΔdndC*), were constructed and provided by Dr. Fen Yao from Prof. Zixin Deng’s lab at Shanghai Jiaotong University, China. All strains were grown in standard Lysogeny broth (LB) medium at 37°C. The cell pellets were collected at the mid-log phase and then, genomic DNA was extracted using E.Z.N.A Bacterial DNA kit (Omega BIO-TEK. United States), following manufacture’s protocol.

### *Salmonella enterica* PCR Sample Preparation for Sequencing

The purified genomic DNA of the three *S. enterica* serovar Cerro 87 DNA samples (wild type, *ΔdndB*, and *ΔdndC*) was used to generate the modification-free reference by PCR amplification; 10 ng of native DNA for each sample was used for amplification using the REPLI-G Kit (Qiagen) according to the manufacturer protocol. The modification-free reference PCR samples were treated with 1 μl of T7 endonuclease (NEB) for 1 h at 37°C to reduce branching derived from the amplification. The samples were cleaned using the DNA Clean and Concentrator Kit (Zymo Research) according to the manufacturer’s protocol.

### *Salmonella enterica* DNA Sequencing

The six samples (native and PCR DNA) were sequenced using the ONT Native barcoding genomic DNA sequencing protocol (SQK-LSK109 and EXP-NBD104); 1 μg of DNA for each of the six samples was used for library preparation according to the manufacturer protocol. Sequencing was performed on a single R9.4 FLO-MIN106 flow cell on a MinION Mk1B for 48 h through MinKNOW software to generate fast5 files.

The sequencing data generated in have been deposited in the Sequence Read Archive database under BioProject PRJNA801225.

### Bioinformatic Analysis

#### Basecalling and Quality Control

The fast5 files were basecalled using Guppy software version 4.4.1 to generate fastq files. NanoFilt software (De Coster et al., 2018) was used to filter only the reads with lengths longer than 200 bp and a mean quality score greater than eight, which were used for further analysis.

#### Identification of PT Modification Sites

We used ELIGOS software (Jenjaroenpun et al., 2020) to identify the DNA modification sites by differential analysis comparing the native DNA sequences of individual samples (plasmid and *S. enterica*) with PT modification-free DNA sequences for all samples. DNA modification sites were identified with an odds ratio cut-off of 1.5 and an adjusted *value of p* of 0.01. The DNA modification sites on the known PT modification motif of 5′-G_PS_AAC-3′/5′-G_PS_TTC-3 were assigned as PT modification sites. To evaluate the disturbances of the ionic signal by PT modification, the raw signals obtained from the fast5 files were resquiggled on the reference sequence using Nanopolish software (Loman et al., 2015). The resquiggle signals were then analyzed using the Box-Cox Transformation, which minimizes the noise from the ONT data. A gene significance score, π-value (Xiao et al., 2014), was used to construct a differential ionic signal following (Nookaew et al., 2020).

#### Genome Base Comparison of PT Modification Sites

We use the ChIPpeakAnno R package (Zhu et al., 2010) to compare the PT modification sites between the samples. The results were summarized in either Venn diagrams or upset plot using the UpSetR R package (Conway et al., 2017). Some selected genes were visualized using the integrative genomics viewer (Robinson et al., 2011).

#### Functional Enrichment Analysis of High-Frequency PT Modification Genes

To identify functional categories of genes in the *S. enterica* serovar Cerro strain 87, all associated sequences were downloaded from GenBank (Sayers et al., 2021). Annotated genes for proteins and RNAs were then searched against the Rfam database (Kalvari et al., 2018, 2021) and InterProscan software (European Molecular Biology Laboratory-European Bioinformatics Institute) using OmicBox (formally Blast2GO) tool version 2.0 (BioBam Bioinformatics, 2021 OmicsBox-Bioinformatics Made Easy). The accession numbers of functional categories were assigned by the most associated hits on the Gene Ontology (GO) database (Mi et al., 2019; The Gene Ontology Consortium, 2021). The GO terms from both proteins and RNAs were then merged and reported based on three categories: biological process (P), cellular component (C), and molecular function (F). The genes that contain PT modifications at a minimum of five sites for individual samples were used for GO Enrichment Analysis using the piano R package (Väremo et al., 2013). These results (enrichment value of *p* < 1e^−5^) were plotted as a heat map.

## Results

### Identification of PT Modifications in the Synthetic 2′Deoxyoligonucleotides and Native DNA Sequences

**Figure 1:**
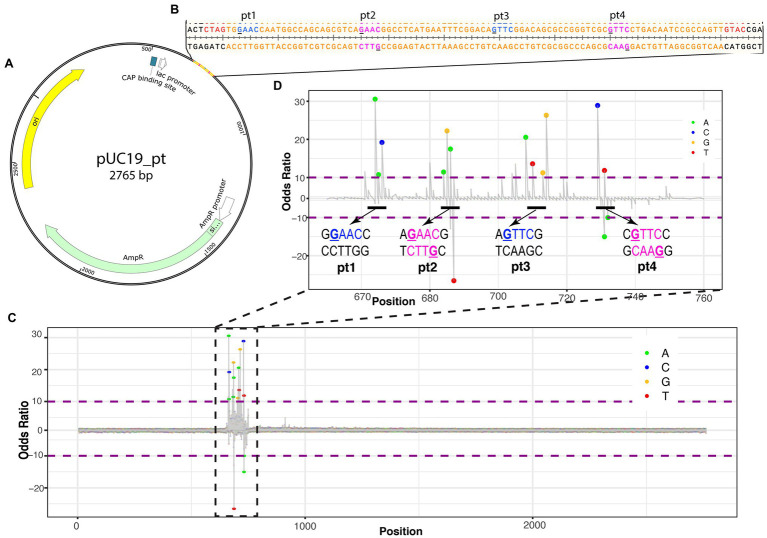
Identifying PT modifications in the pUC19 plasmid. **(A)** Schematic of pUC19_pt plasmid with genes (AmpR in green, origin in yellow, and CAP binding site in white) and 2′deoxyoligonucleotide with PT modifications insert. **(B)** Nucleotide sequence for insert (in orange). PT modification sites were added at the underlined guanines of the insert. Blue nucleotides are single-stranded PT modifications; magenta nucleotides are double-stranded PT modifications. Red nucleotides are the sticky ends that ligate into Xbal and Kpn1 cut sites of the plasmid and are included on the insert. **(C)** ELIGOS-determined odds ratio at each base within the plasmid showing the highest odds ratios at the insert. **(D)** Odds ratio of the insert showing spikes around the manufactured PT modifications. The bases within the odds ratio range from 10 to −10 are identified with a dot color coded to each base (see color key in top right corner of the panel). The dots will be shown when adjusted to value of *p* < 10e-50.

Nucleic acid modifications can be identified from native sequences using the ONT platform by comparing the signals of modification-free control DNA bases to the signals with modifications (Jenjaroenpun et al., 2020; Nookaew et al., 2020). To establish the ONT platform sequencing capability to identify PT modifications, a plasmid with a synthetic 2′deoxyoligonucleotide insert containing the PT modifications was sequenced (Figure 1A). The 2′deoxyoligonucleotide was designed with sticky ends to ligate into XbaI and KpnI cut sites in pUC19, and four PT modification sites were added, with two sites being single-stranded modifications and two being double-stranded modifications (Figure 1B).

ELIGOS calculates the error at specific base and odds ratio (comparison of pUC19_pt plasmid with the modification-free control plasmid) from the sequencing reads at each position on the pUC19_pt plasmid and the modification-free control plasmid. As the sequence approaches the synthetic 2′deoxyoligonucleotide insert, the odds ratio values significantly differ from the modification-free control plasmid (Figure 1C).

As we zoom in on the odds ratio for positions 670–760, the specific bases with significantly different errors become evident. Six to seven nucleotides are within the nanopore as each base is read. DNA modifications cause disturbances while entering, passing through, and exiting the nanopore affecting the signal of neighboring bases present in the pore at the time of reading (Nookaew et al., 2020); this is evident with the synthetic PT modifications as the odds ratio spike for numerous bases around the modified positions (Figure 1D).

### Characterization of PT Modification Disturbances to Ionic Signal

**Figure 2:**
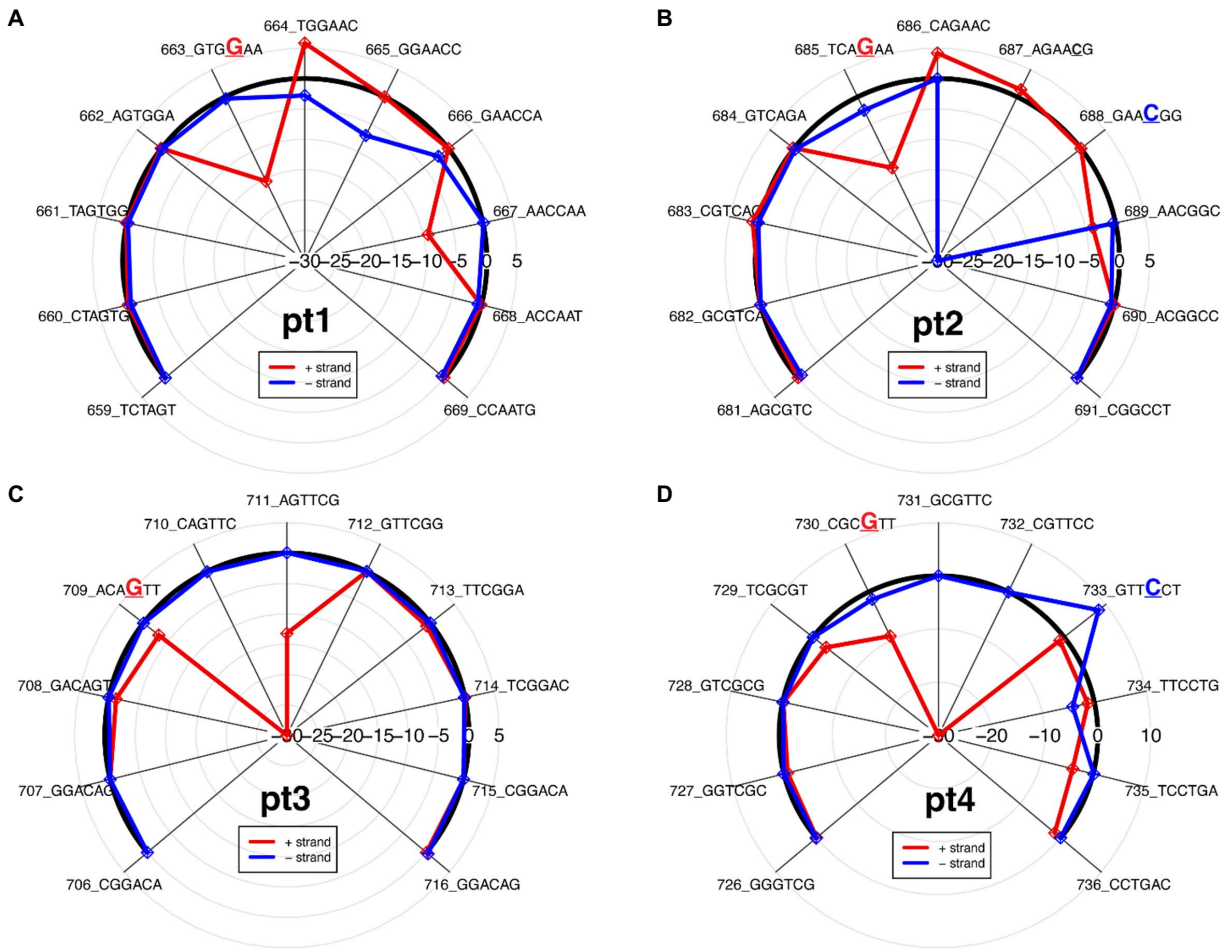
Differential ionic signal radar plots, which is derived from resquiggle signals, for each PT modification site as seen on the 2′deoxyoligonucleotide insert in Figure 1. **(A)** Pt1, single-stranded modification on positive strand; **(B)** Pt2, double-stranded modification; **(C)** Pt3, single-stranded modification on positive strand; and **(D)** Pt4, double-stranded modification.

The ionic signal of the ONT platform can be used to determine which bases pass through the nanopore during sequencing and, in the case of modified bases, which errors occur. The differential changes in the pUC19_pt and modification-free control plasmid’s ionic signal can be observed based on the differential ionic signal plot. The change in the ionic signal can be positive, an increased ionic signal, or negative, a decreased ionic signal, as compared to the modification-free control plasmid (Figure 2).

In general, PT modifications affect signal alteration at multiple positions and the signature of ionic signal changes depends on the sequence context surrounding the modified base, as previously observed (Jenjaroenpun et al., 2020; Nookaew et al., 2020). While the PT modification for pt1 and pt3 is only on the positive strand, compared to the modification-free control plasmid, the varying ionic signal on pt1 indicates that both strands are affected between kmers 662 and 668 (Figure 2A), whereas the ionic impedance on pt3 indicates that only the positive strand is affected as the π statistic dips down to −30 at the kmer containing the modified guanine (Figure 2C).

The double-stranded PT modifications showed ionic disturbances on both strands, but only one strand showed greater impedance. Compared to the modification-free control plasmid, pt2 shows a greater disturbance on the negative strand, as indicated by a greatly decreased ionic signal between kmers 686 and 689 (Figure 2B), whereas pt4 shows a greater disturbance on the positive strand, as indicated by a greatly decreased ionic signal between kmers 736 and 733 (Figure 2D). The results agree with the odds ratio plot and show that the ONT/ELIGOS platform correctly identifies and localizes PT modifications.

### Identification of PT Modifications in *Salmonella enterica* Native DNA Sequences

**Figure 3:**
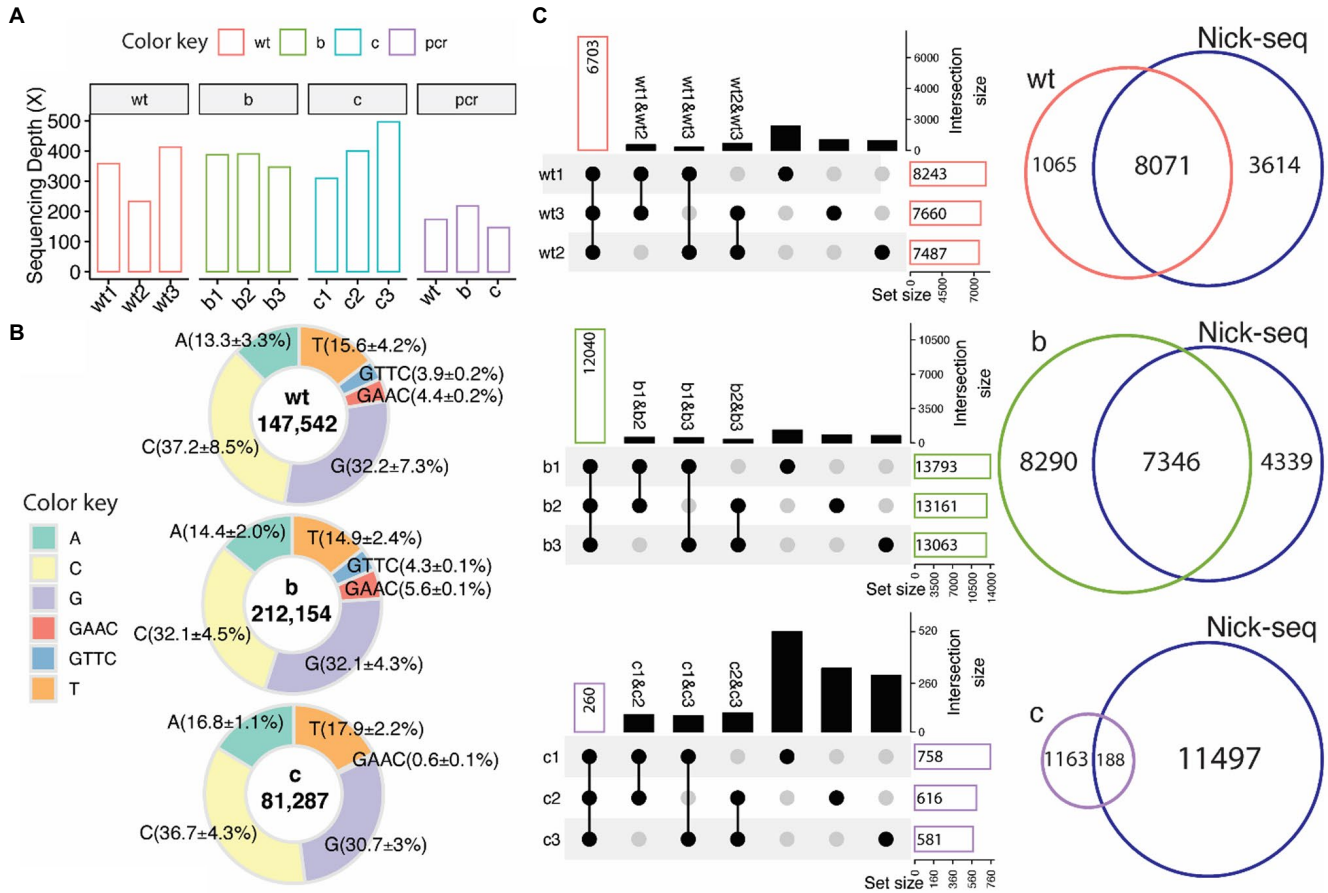
PT modifications in *Salmonella enterica* from native DNA sequences. **(A)** Sequencing depth for wild type (wt), ∆*dndB* **(B)**, ∆*dndC* **(C)**, and modification-free reference PCR DNA from each strain (PCR). **(B)** Percentage distribution of modification sites of each base (A,T,C,G) and the two PT motifs (GAAC/GTTC, backbone modifications classified by adjacent base) compared to the modification-free reference PCR DNA. ELIGOS determination of DNA modification sites (number inside the pie graph) including PT modification sites (GAAC/GTTC) within wild type, ∆*dndB*, and ∆*dndC* strains of *S. enterica*. **(C)** Upset plots (left panel), which compose of overlap matrix in dot plot with the overlap size in bar plots, show overlap of PT modification sites identified between replicates. The black dots represent the overlap of PT sites among samples connected with black lines. The bar plots reflect the overlap of detected PT modification sites in the selected replicates and the absence of a PT modification in the de-selected replicate. The gray dots represent no overlap found. The Venn diagram (right panels) shows overlap of PT modification sites identified by the ONT platform and Nick-seq data of *S. enterica* wild-type strain.

The ONT/ELIGOS platform was then used to identify PT modifications in three strains of *S. enterica* (wild type, *ΔdndB*, and *ΔdndC,* see Supplementary Figure S3 for gene deletion confirmation). The sequencing depth for the three replicates from each strain and the coverage for the modification-free, amplified DNA (PCR) for the three strains were analyzed (Figure 3A). For the wild-type strain, the sequencing depth ranges from 200x to 400x, while the two mutant strains range from 350× to 400× for *ΔdndB* and 300× to 500× for *ΔdndC*.

As previously reported by He et al. (2015) and Chao et al. (2017), the predominant PT modification motifs for *S. enterica* are GTTC and GAAC, representing double-stranded PT modification sites with PT between GT for GTTC and GA for GAAC. We calculated the percentage of modifications for each base and the two PT motifs compared to the modification-free PCR DNA (Figure 3B). Guanine and cytosine are the most modified bases across all three strains, with 3.9% of the GTTC and 4.4% of the GAAC motifs being modified in the wild-type strain compared to the modification-free, PCR DNA. These percentages increase to 4.3% for GTTC and 5.6% for GAAC in the ∆*dndB* mutant strain and decrease to nondetectable for GTTC and 0.6% for GAAC in the ∆*dndC* strain.

Three replicates for each strain were sequenced to establish consistency with the technique. Figure 3C shows the intersection size of the three replicates per strain. The ∆*dndB* strain has the most PT modification sites and the largest intersection size across replicates, while ∆*dndC* has the least amount of PT modification sites and the smallest intersection size. We also compared our data to the Nick-seq PT modification site data derived from *S. enterica* wild-type strain (Cao et al., 2014). The wild-type and ∆*dndB* strains showed vast overlap in PT modification sites between the two techniques, while the decreased PT modification sites in the ∆*dndC* showed less overlap with the Nick-seq data (Cao et al., 2014). The Nick-seq/SMRT sequencing techniques showed PT modifications at about 10% of the available motifs (Cao et al., 2020).

### PT Modification Sites Per Gene Within *Salmonella enterica*

**Figure 4:**
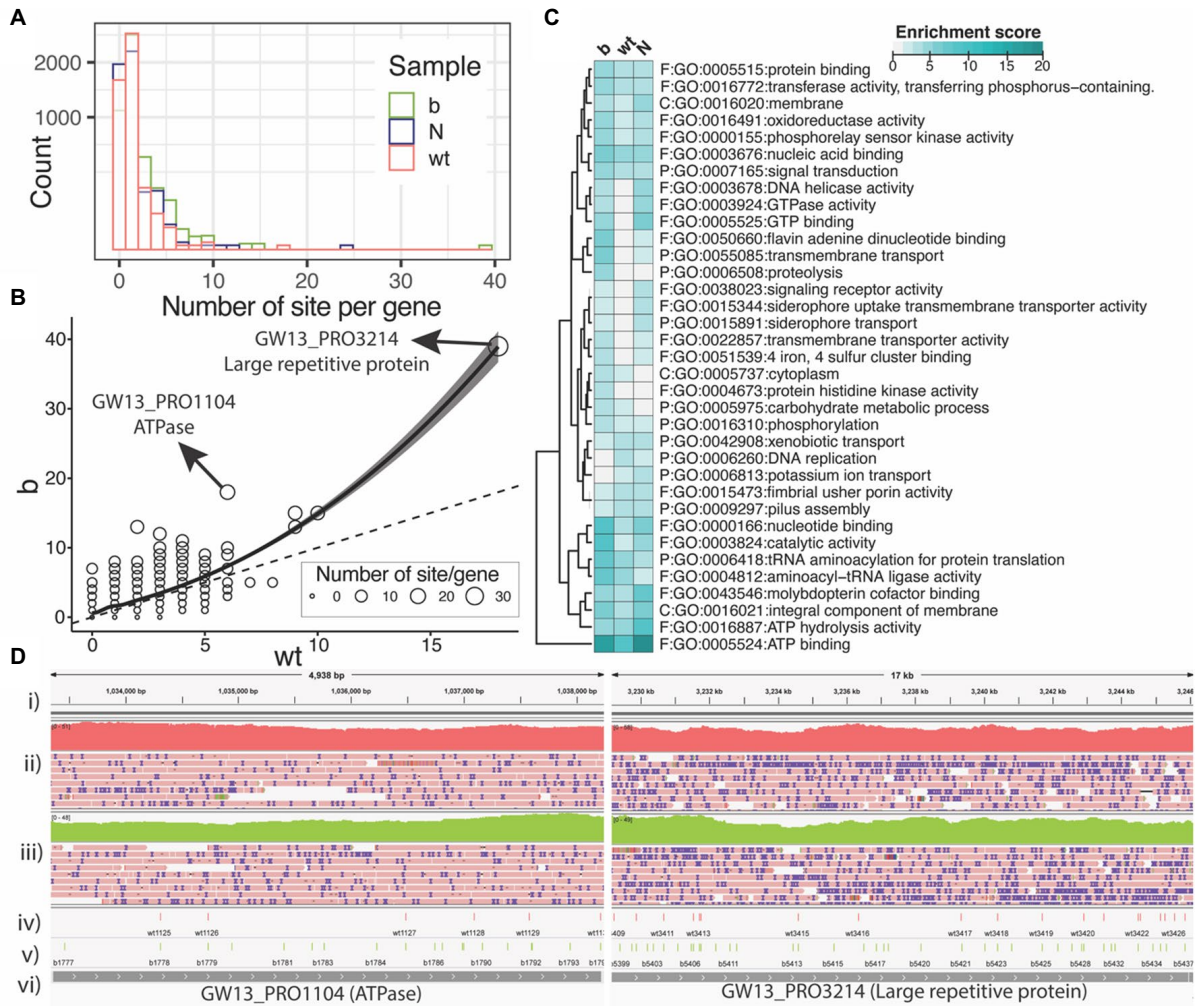
PT modification sites within the *Salmonella enterica* genome. **(A)** Number of PT modification sites per gene (square root *y*-axis) in the three samples of wild type (wt), ∆dndB **(B)**, and the Nick-seq data (N). **(B)** Number of PT modification sites per gene for the wild-type strain vs. ∆dndB strain. **(C)** List of genes with the most PT modification sites per strain. **(D)** Integrative Genomics Viewer images for ATPase and Large repetitive protein genes; (i) reference genome, (ii) wild-type gene coverage and reads, (iii) ∆dndB gene coverage and reads, (iv) wild-type PT modifications, (v) ∆dndB PT modifications, and (vi) gene names.

Finally, we wanted to explore where within the *S. enterica* genome these modifications occur. Figure 4A is a histogram showing the number of PT modification sites per gene for each of the three strains. Many of the genes have only one or two PT modification sites per gene. However, a few genes have five or more PT modifications. The ∆*dndB* strain shows more PT modification sites per gene than the wild type (Figure 4B). In addition, we observed a very small number of 200 bp upstream start codons contained PT sites (see Supplementary Figure S4).

We next summarized the functional enrichment of the genes with at least five PT sites per gene for each sample of wild-type and ∆*dndB* strains with the data from the Nick-seq as shown in Figure 4C. The most modified gene across all three strains is the ATP binding genes. Interestingly, the wild-type strain showed downregulation of PT modification sites in many of the genes listed compared to the ∆*dndB* (increased modification) strain and the ∆*dndC* (decreased modification) strain.

The distribution (Figure 4D) of PT modifications for wild-type strain and ∆*dndB* strain within the ATPase and large repetitive protein genes, which have the most PT modification sites per gene in the ∆*dndB* strain. For ATPase, ∆*dndB* has 17 PT modifications, while the wild type has only six. The ∆*dndB* strain has 39 PT modifications within the large repetitive protein gene, while the wild type has 19.

## Discussion

Here, we report the application of the ONT platform coupled with the ELIGOS tool for identification of PT modifications *in vitro* and *in vivo*. We established the method using double-stranded oligonucleotides containing known PT modification sites. When comparing the modified 2′deoxyoligonucleotide DNA (pUC19_pt) to modification-free DNA (pUC19), ELIGOS was able to identify the PT modification sites (Figures 1, 2). PT modifications show a cross-strand disturbances of the ionic signal (Figure 2). Further research is needed to elucidate the cause.

The method was applied to genomic samples using *S. enterica.* To identify PT modification sites *in vivo*, we used a wild-type strain; ∆*dndB*, a negative regulator knockout strain known to increase PT modifications; and ∆*dndC*, a sulfurtransferase knockout strain known to decrease PT modifications. PT modification sites were identified in the three strains, with ∆*dndB* having the most sites, and ∆*dndC* having the fewest sites that agree with the known regulation of PT modification in *S. enterica* (Figures 3, 4). The relatively small amount of PT modifications sites detected with a low fraction of overlap among the replicates in Figure 2C bottom panel was possibly derived from the sulfurtransferase activity of other genes as summarized in Supplementary Table S1. Many of the genes with upregulated PT sites are genes involved with ATP utilization. The proteins encoded by these genes are important for cellular redox homeostasis needed for survival; therefore, it would benefit from protection from bacteriophage attack by increase PT modification. The regulation of PT modification could impact the gene expression pattern in the *S. enterica* as observed in protein abundance (He et al., 2015). This is an important research area that needs further investigation. The results show that the ONT platform can accurately detect PT modifications in native DNA sequences, compared to previous methods used for detection, with high reproducibility.

One of the limitations of this study is that the method involves determining PT modification sites based on the error at that nucleotide. The ONT platform has a higher error rate than next-generation sequencing techniques; however, modified nucleotides can be distinguished from background error rates with enough read coverage. Chao et al. reported the common substrates of GATC/CTAG and GAAC/GTTC for methyltransferase and sulfurtransferase in the generation of m6A modification, PT modification, and hybrid modification of the substrates (Chao et al., 2017). Therefore, using GAAC/GTTC with error profiles to flag PT modifications for detection is a limitation of this study. To overcome these limitations, the analysis of raw signals such as deep learning is needed to refine the identification of PT modification that will need gold standard datasets for model training. Another limitation is that we only sequenced one species containing the *dnd* operon. The few non-overlapped PT modification sites observed may be derived from a different culture of the *S. enterica* leading to different regulation of PT modifications that need further deep investigation. For future studies, we would like to apply the method to other species known to have PT modifications to better understand the PT modifications of bacteria.

In conclusion, the ONT platform is suitable for identification of PT modifications in native DNA sequences. This method can also identify the loci of the PT modifications within genomes. This method is expected to be suitable to study PT modifications in other species and various sequence contexts.

## Data Availability Statement

The datasets presented in this study can be found in online repositories. The names of the repository/repositories and accession number can be found at: https://www.ncbi.nlm.nih.gov/genbank/, PRJNA801225.

## Author Contributions

IN designed and conceived the project. GB and PD participated in experimental design, set up, project planning, and manuscript development. TW and SM performed the synthetic plasmid constructions with the advice of EH. MD performed the cell culture and DNA purification. TW performed all sequencing. IN and TW performed the data analysis and wrote the manuscript draft. All authors contributed to the article and approved the submitted version.

## Funding

This work supported by the National Institute of General Medical Sciences of the National Institutes of Health (P20GM125503 to IN). This material is based upon work supported by the National Science Foundation under Award no. OIA-1946391 and the National Institute of Environmental Health Sciences of the National Institutes of Health (R01-ES031576 to PD).

## Conflict of Interest

The authors declare that the research was conducted in the absence of any commercial or financial relationships that could be construed as a potential conflict of interest.

## Publisher’s Note

All claims expressed in this article are solely those of the authors and do not necessarily represent those of their affiliated organizations, or those of the publisher, the editors and the reviewers. Any product that may be evaluated in this article, or claim that may be made by its manufacturer, is not guaranteed or endorsed by the publisher.
