# Supplementary material for: Nanopore Sequencing for Detection and Characterization of Phosphorothioate Modifications in Native DNA Sequences
Source: Front Microbiol. 2022 Apr 21;13:871937. doi: 10.3389/fmicb.2022.871937 (PMC9069010; doi:10.3389/fmicb.2022.871937)
Supplement: Supplementary file 1 [file Data_Sheet_1.PDF]

Supplementary Infomation

CTAGTGGAACCAATGGCCAGCAGCGTCAGAACGGCCTCATGAATTTCGGACAGTTCGGACAGCGCCGGGTCGCGTTCCTGACAATCCGCCAGTTGTAC

ACCTTGGTTACCGGTCGTCGCAGTCTTGCCGGAGTACTTAAAGCCTGTCAAGCCTGTCGCGGCCCAGCGCAAGGACTGTTAGGCGGTCAA

Supplementary Figure 1. Nucleotide sequence for synthetic PT oligonucleotide. Red nucleotides are the insert site complementary with sticky end cut of sites XbaI and KpnI on pUC19 plasmid. Blue nucleotides are single-stranded modification sites. Magenta nucleotides are double-stranded modification sites. The PT modified nucleotides are underlined.

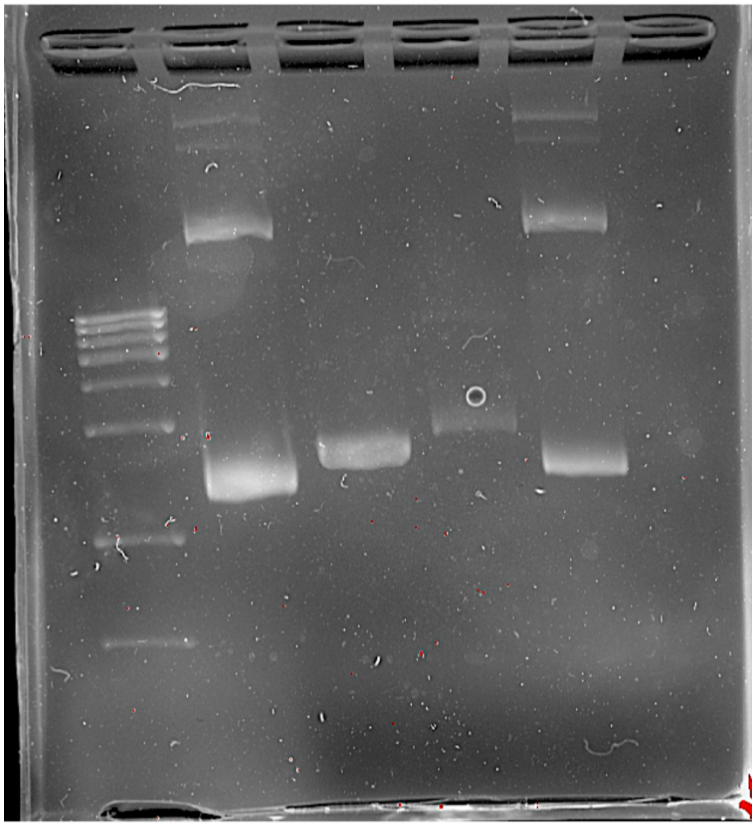

Supplementary Figure 2. 2% agarose gel. Lane one is a 1 kb ladder. Lane two is pUC-19 plasmid. Lane three is pUC-19 plasmid cut by restriction enzymes. Lane 4 is pUC-19 plasmid with ligated PT oligonucleotide insert. Lane five is transformed pUC-19 plasmid with PT oligonucleotide insert.

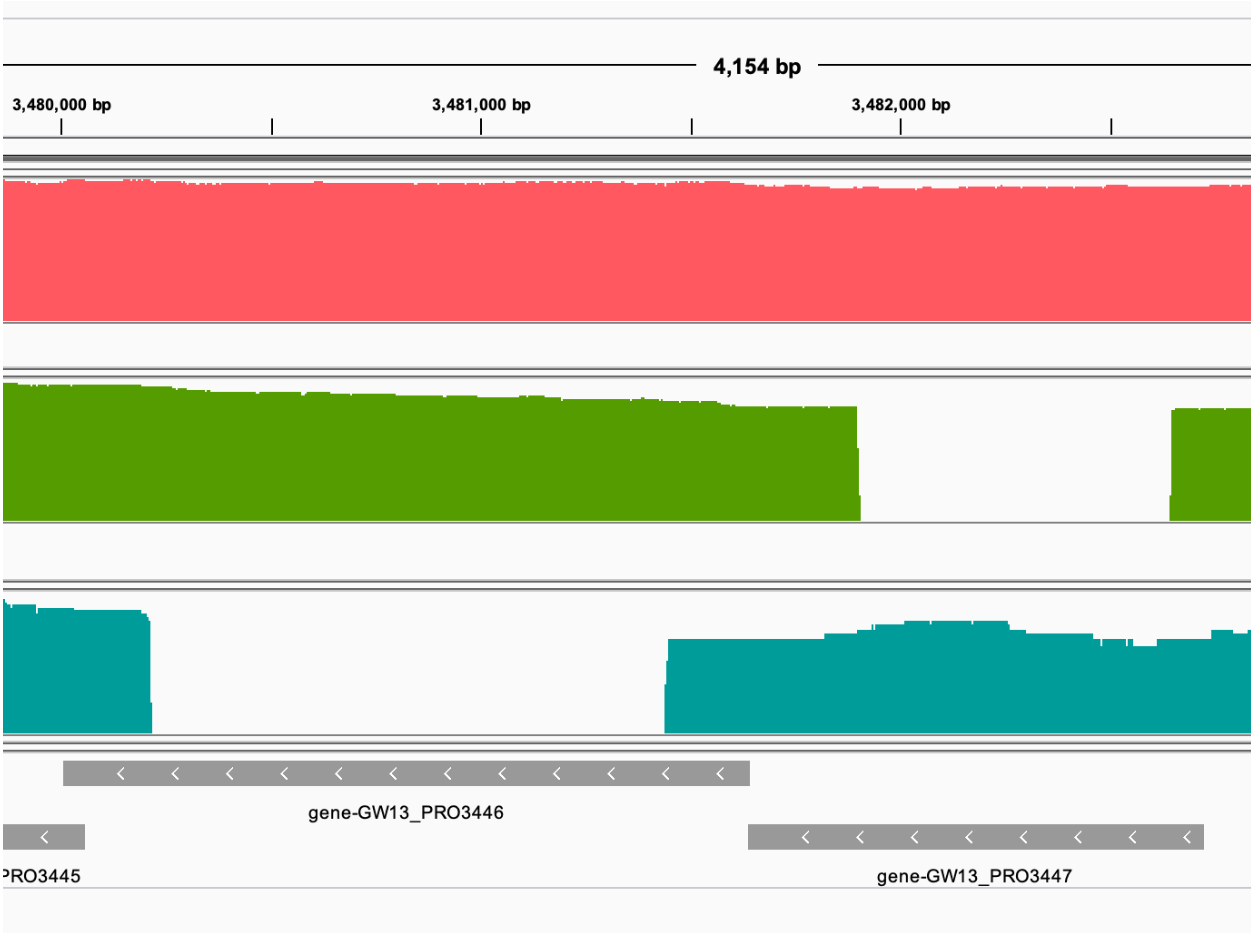

Supplementary Figure 3. IGV show the read coverage over *dndB* (GW13_PRO3446) and *dndC* (GW13_PRO3447) of *S. enterica* wild-type (red), Δ*dndC* (green) and Δ*dndB*(teal).

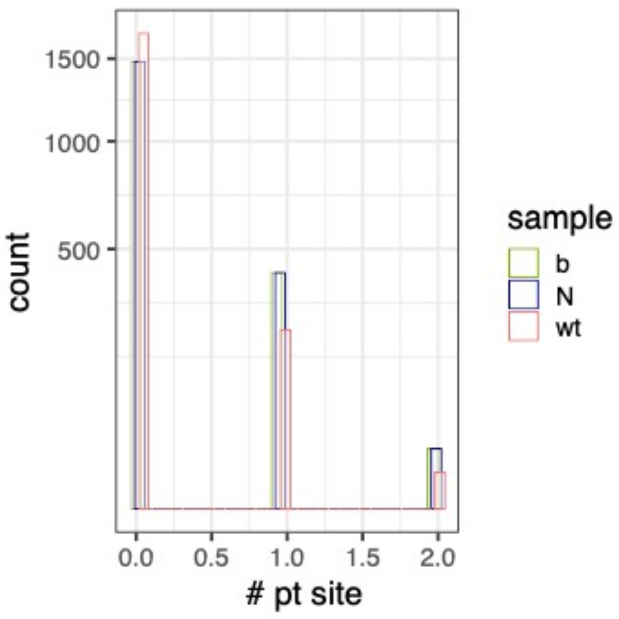

Supplementary Figure 4. Histogram shows number of PT site(s) (square root yaxis) of 200 bp upstream start codons.

Table S1. Genes that have sulfurtransferase activity

accession number (NCBI)	Locus tag	name	Flag
APT78501.1	GW13_PRO1625	Thiosulfate sulfurtransferase, rhodanese	
APT79407.1	GW13_PRO2531	Thiosulfate sulfurtransferase GlpE	
APT79577.1	GW13_PRO2701	sulfurtransferase	
APT78643.1	GW13_PRO1767	sulfurtransferase	
APT77939.1	GW13_PRO1063	sulfurtransferase	
APT80318.1	GW13_PRO3446	3'-phosphoadenosine 5'-phosphosulfate sulfurtransferase	dndC
